# What Makes a Protein Sequence a Prion?

**DOI:** 10.1371/journal.pcbi.1004013

**Published:** 2015-01-08

**Authors:** Raimon Sabate, Frederic Rousseau, Joost Schymkowitz, Salvador Ventura

**Affiliations:** 1 Departament de Fisicoquímica, Facultat de Farmàcia, Universitat de Barcelona, Barcelona, Spain; 2 Institut de Nanociència i Nanotecnologia (IN2UB), Universitat de Barcelona, Barcelona, Spain; 3 VIB Switch Laboratory, VIB, Leuven, Belgium; 4 Departement for Cellular and Molecular Medicine, KU Leuven, Leuven, Belgium; 5 Institut de Biotecnologia i de Biomedicina, Universitat Autònoma de Barcelona, Bellaterra, Spain; 6 Departament de Bioquímica i Biologia Molecular, Universitat Autònoma de Barcelona, Bellaterra, Spain; Wake Forest University, United States of America

## Abstract

Typical amyloid diseases such as Alzheimer's and Parkinson's were thought to exclusively result from *de novo* aggregation, but recently it was shown that amyloids formed in one cell can cross-seed aggregation in other cells, following a prion-like mechanism. Despite the large experimental effort devoted to understanding the phenomenon of prion transmissibility, it is still poorly understood how this property is encoded in the primary sequence. In many cases, prion structural conversion is driven by the presence of relatively large glutamine/asparagine (Q/N) enriched segments. Several studies suggest that it is the amino acid composition of these regions rather than their specific sequence that accounts for their priogenicity. However, our analysis indicates that it is instead the presence and potency of specific short amyloid-prone sequences that occur within intrinsically disordered Q/N-rich regions that determine their prion behaviour, modulated by the structural and compositional context. This provides a basis for the accurate identification and evaluation of prion candidate sequences in proteomes in the context of a unified framework for amyloid formation and prion propagation.

## Author Summary

Protein conformational disorders include several neurodegenerative diseases. These pathologies are initiated by conformational changes in specific polypeptides that, in many cases, result in their spontaneous self-assembly to form toxic amyloids. Prions are a subclass of amyloids with the ability to propagate *in vivo*, thus becoming infectious. Previous work with yeast prions has provided tremendous insight into prion propagation mechanism. These proteins contain glutamine/asparagine (Q/N) enriched prion forming domains (PFDs), which are both necessary and sufficient for propagation. We found that these domains include specific short amyloid-prone sequences, which are likely able to trigger the amyloid conversion of the complete prion protein. The amyloid potency of these short segments suffices to discriminate with high accuracy between Q/N rich domains with and without prion activity. Our data suggest a model for prions where a classical amyloid core is embedded in a sequence context that reduces the amyloid nucleation potential, resulting in sequences that are strongly dependent on seeding. This model should allow the identification of prion-like proteins in the human proteome and prediction of the deleterious effects of genetic mutations occurring in these particular proteins.

## Introduction

Amyloid structures are associated with an increasing number of human disorders [1]. Prions have been considered a particular subclass of amyloids in which the aggregation process self-perpetuates *in vivo*, thus becoming infectious. However, increasing evidence suggests that *in vivo* protein cross-seeding may in fact reach beyond the scope of relatively rare disorders such as Kuru or Creutzfeld-Jacob Disease, to frequently occurring neurodegenerative pathologies, including Alzheimer's and Parkinson's diseases [2]–[4]. So far it seems that the similarity only covers certain aspects of prion behaviour, causing Aguzzi to coin the term 'prionoids' [5]. A unifying aspect is certainly the amyloid structure adopted by prions and prionoids alike and which is thought to be behind *in vivo* seeded aggregation. However, the critical features that allow a specific amyloidogenic sequence to become prionogenic and thus infectious are still not clear.

Fungal prions provide excellent model systems for the understanding of amyloid formation and propagation [6]. An increasing number of prion proteins are being identified in yeast, the best-characterized being NEW1, RNQ1, SWI1, SUP35 and URE2 proteins. Protein domains involved in prion formation in all these polypeptides are highly enriched in asparagine (N) and/or glutamine (Q) residues and often correspond to intrinsically unstructured protein regions. Protein domains displaying this sequence signature are over represented in eukaryotic genomes relative to prokaryotes. Given their potential involvement in pathogenic processes, the fact that these sequences have not been suppressed by purifying selection suggests that prion-like conformational conversion may have evolved as a mechanism for regulating functionality in eukaryotic proteins [7]. Recently, Lindquist's group conducted a genome-wide *in silico* survey to identify prionogenic proteins in the *S. cerevisiae* proteome on the basis of their compositional similarity to known prion forming domains (PFDs) using a hidden Markov model. The prionogenic nature of the top 100 identified candidate PFDs was evaluated through experimental investigations of four *in vitro* and *in vivo* prion characteristics [8]. 29 of them, including the PFDs of previously known yeast prions, showed one of the key features, namely switching behaviour between a soluble and prion form *in cells* or strong amyloid formation capability. Nevertheless, still 68% of the fully characterized domains turned to be false-positives, even with several of them displaying the highest composition similarity to known PFDs. This dataset provides an outstanding benchmark to decipher how prion transmissibility is encoded in polypeptide sequence.

The sequence of a protein determines to a large extent its amyloid propensity [9]. It has been argued, however, that two distinct classes of amyloid polypeptides exist [10]. The first class follows the by now classical short-stretch model [11]–[13], in which self-assembly is thought to be nucleated specifically by short sequences of high amyloid propensity, whereas in the second class a large number of weak interactions between side-chains in large, structurally disordered domains would constitute the driving force for amyloid formation. According to this classification, prionoid proteins like Aß, tau and α-synuclein would belong to the first class, whereas the Q/N rich yeast prions will fall in the second one. It has been suggested that this mechanistic difference would explain why algorithms designed to detect short and specific amyloid motifs in proteins [14] usually fail to classify correctly Q/N rich prionic and non-prionic sequences [15].

The WALTZ algorithm [16] (http://waltz.switchlab.org/) uses a position-specific scoring matrix deduced from the biophysical and structural analysis of the amyloid properties of a large set of hexapeptides (S1 Fig.). A distinctive feature of WALTZ is that most amino acids are predicted to display differential amyloid propensities depending on their specific position in the sequence. This is the case of Q and N residues, which contribute positively or negatively to the amyloid potential depending on their position (S1 Fig.). The edge positions 1, 2 and 6 in the matrix display low selectivity, whereas the core positions 3, 4 and 5 are highly restrictive. Aromatic and hydrophobic residues remain most favored in the core positions. However, certain polar residues, including Q and N, can be accommodated or even be favorable in these locations. The ability to consider position-specificities allows WALTZ to specifically identify short sequences leading to ordered amyloid aggregates, including those formed by Q/N enriched SUP35 decapeptides [16]. Here we show that this ability can be exploited to classify prionic and non-prionic sequences providing an alternative description of the sequence features that underlie prion formation. In our model, prionogenic behaviour requires the embedding of a relatively short amyloid forming sequence in a flexible region enriched in the typical polar amino acids Q and N. This alternative model of prion behavior provides a unified framework for amyloid formation and prion propagation.

## Results

### The relationship between amyloidogenicity and prion propensity

Ross and Toombs have shown that the sequence of a short eight-residue stretch of a variant of Sup35 PFD suffices to determine the priogenicity of the complete protein, revealing that the presence of hydrophobic residues, which are otherwise under-represented in PFDs, highly increase the overall prion propensity [17]. The presence of hydrophobic residues is recurrently observed in amyloid sequences and, in fact, when we analysed the 62 sequence variants they tested in this short Sup35 region using WALTZ (with default settings) we observed that 44.4% of prion-promoting sequences were predicted as amyloidogenic, whereas only 14.2% of non-prionic sequences were identified as such (S1 Table). This suggests that the enrichment in hydrophobic residues in prion-promoting stretches acts by increasing their sequential amyloid propensity and therefore that the presence of short and specific amyloid sequences might be an important contributor to the prionogenicity of a Q/N rich sequence, as previously proposed [18], [19]. Based on this hypothesis, we wondered if prediction of amyloidogenicity might aid to discriminate prion from non-prions in the protein dataset experimentally characterized by Alberti et al. The authors of that study scored the domains from 0 to 10 according to their combined performance in four different assays that include tests for both amyloid and prion forming ability. We considered as non-prions those sequences scoring ≤2 and being positive in one assay at maximum, meaning that they do not exhibit amyloid and prion forming ability at the same time, yielding a total of 39 sequences (Table 1). We considered as prions those domains being positive in all four assays and scoring ≥9, with a total of 12 sequences, including the known prions NEW1, RNQ1, SWI1, SUP35 and URE2 proteins (Table 1).

**Table 1:** Prediction of the prionic behaviour of putative Q/N rich yeast prions.

Gene Name	pWALTZ Score	P/NP (b)	Gene Name	pWALTZ Score	P/NP (b)
MCM1	59.63	NP	PUF2	73.07	P
NAB2	56.43	NP	SWI1	75.43	P
TAF12	55.60	NP	KSP1	75.97	P
YCK1	64.05	NP	ASM4	75.73	P
MED2	65.49	NP	URE2	73.66	P
AKL1	69.90	NP	GLN3	74.36	P
PUF4	73.85	NP	RNQ1	74.82	P
PCF11	64.29	NP	NEW1	85.60	P
EPL1	68.82	NP	NRP1	76.11	P
SNF2	67.91	NP	LSM4	76.89	P
SCD6	67.03	NP	YBL081W	76.78	P
YAK1	61.84	NP	SUP35	73.99	P
CAF40	56.70	NP			
NRD1	56.92	NP			
PDC2	68.66	NP			
RAT1	71.46	NP			
SLA1	68.05	NP			
SIN3	69.95	NP			
UPC2	73.44	NP			
TIF4632	71.64	NP			
CLA4	53.86	NP			
SKG3	68.47	NP			
TIF4631	66.13	NP			
SLT2	51.68	NP			
AZF1	72.27	NP			
CCR4	65.89	NP			
NUP57	73.01	NP			
SSD1	69.09	NP			
VTS1	69.76	NP			
PSP1	73.05	NP			
YAP1802	64.13	NP			
YMR124W	53.75	NP			
*ENT2*	-	NP			
*SKG6*	-	NP			
*YLR177W*	-	NP			
*PIN3*	-	NP			
*WWM1*	-	NP			
*NAB3*	-	NP			
*HRR25*	-	NP			

Prion recovery using the prion/non-prion (NP/P) classification of selected putative prions according to Alberti's scale of prion activity. Sequences with pWALTZ score >73.55 are considered prion-like. False positives and false negatives are underlined. Proteins devoid of any predicted amyloid core are shown in italics (SUP35, in plain text, was not included in the test set to avoid overlap with the WALTZ training set).

(b) Prion/Non-Prion (P/NP) classification according to Alberti et. al. scale of prion propensity (*Cell* 137, 146–158). Sequences scoring ≤2 (1 positive assay as a maximum) are considered non-prions (NP) while sequences scoring ≥9 (all four asays positives) are considered prions (P).

The unique atomic-resolution structure of an infectious fibrillar state to date corresponds to HET-s PFD of the fungus *Podospora anserina*. In its fibrillar conformation, HET-s PFD forms a left-handed β-solenoid, with each molecule forming two helical windings [20]. The two repeating strand–turn–strand motifs (β1–β2 and β3–β4) forming two turns of the solenoid contain 21 residues each. A distant homolog of the fungal HET-s prion in *Fusarium graminum* adopts an analogous structure with strand–turn–strand motifs of ∼21 residues [21]. Although the HET-s PFDs are not related to those found in yeast, this implies that a length of 21 might suffice to form a transmissible β-fold. Therefore, we used a 21 residues sliding window to analyse the amyloid propensity of the complete PFD sequences in our dataset, with sizes comprised between 60 and 385 residues, with WALTZ. In addition, because Pro has been shown to act as a β-breaker residue [22] that efficiently opposes β-aggregation [23], [24] and mutation of any residue in HET-s PFD β-strands to Pro abrogates prion propagation [25], we defined that the 21 residues windows cannot include Pro residues.

**Figure 1:**
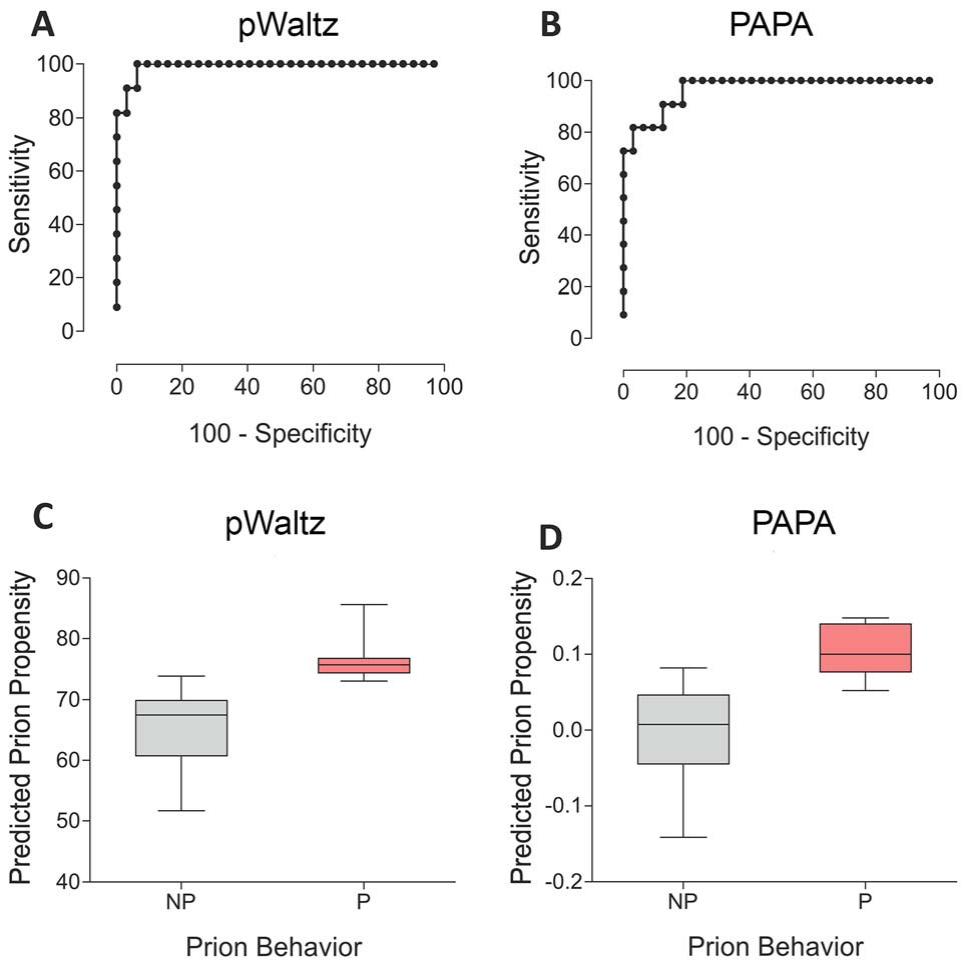
Prediction of prion propensity of Q/N-rich putative prions. Prion recovery using the prion/non-prion (NP/P) classification of selected putative prions according to Alberti's scale of prion activity. ROC plots shows pWALTZ (A) and PAPA (B) performance and box plots showing predicted prion propensity as scored by pWALTZ (C) and PAPA (D) are shown.

**Figure 2:**
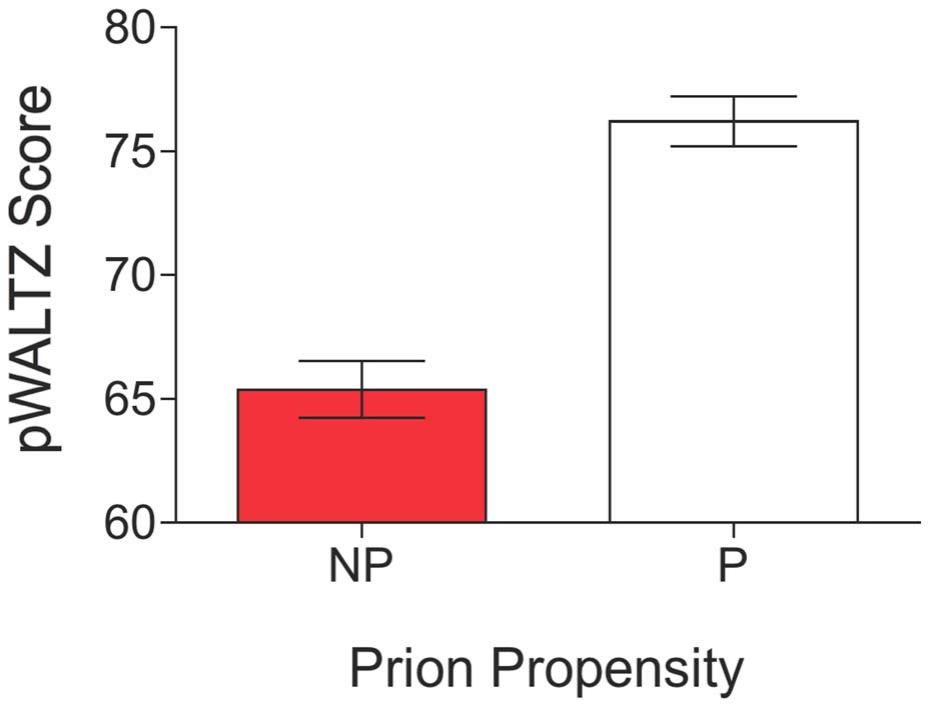
Relationship between amyloid and prion propensities. Average pWALTZ scores of prion (white) and non-prion (red) domains.

The WALTZ algorithm can be run using different levels of stringency or custom defined thresholds. In a typical use, WALTZ high stringency levels (>90%) are employed in order to identify very short and potent segments able to nucleate amyloid formation with high specificity. For example, the analysis of the 758 residue long Tau protein renders a single prediction overlapping with the experimentally validated hexapeptide 591-KVQIIN-596 [26]–[31]. However, the identification and scoring of these strong and short protein stretches, usually flanked by highly soluble residues, does not allow an accurate discrimination between prionic and non-prionic Q/N rich sequences [15]. In the present approach, a sequence is considered for further analysis as a putative PFD candidate only if at least in one of the sliding windows all the 21 residues display values higher than the a given threshold. SUP35 was excluded from the test set, since one tetra- and three hexa-peptides belonging to its PFD sequence were part of the WALTZ training set. We used receiver operating characteristic (ROC) curves and evaluated the area under the curve (AUC) for each particular WALTZ stringency level (between 0 and 100%) and used the derived Youden's index for each plot to identify the threshold and the associated WALTZ score rendering the best predictability. The best values were obtained using a threshold of 35%. Despite this amyloidogenicity value is very low, according to the WALTZ scale, already seven of the non-prion proteins did not exhibit any continuous 21 residues sequence stretch able to pass the threshold. We used the rest of 32 non-prion domains and the 11 prion domains to elaborate the correspondent ROC plot which displays a striking AUC of 0.99 (Fig. 1), employing a WALTZ score cut off of 73.55% to discriminate between prion and non-prion domains (Table 1) according to the associated Youden's index. With these parameters, the approach, which we call now as pWALTZ, has a significance P value <0.0001, a sensitivity of 90.9% and a specificity of 97.4%, with only one false positive (PUF4) and one false negative (PUF2) among the 43 analysed proteins (Table 1) and an overall accuracy of 95.3% (Fig. 1). All the known bona fide yeast prions included in the test set (NEW1, RNQ1, SWI1 and URE2) are correctly classified as positive hits. The approach outperforms composition based algorithms like PAPA (Fig. 1) [15], which displays a 86.0% overall accuracy in the same dataset. As expected, SUP35 is also correctly classified as a prion (Table 1). As shown in Fig. 2, prionic sequences display clearly overall higher pWALTZ values than non-prionic ones. The observed difference is significant, especially if we take into account that we do not include in the comparison those sequences that failed to past the soft 35% initial threshold. An example of the scoring of prion and non-prion sequences is provided in the Supplementary Material (S2 Fig.).

In a pioneering exercise, Ross and co-workers used PAPA to design two synthetic PFD (s-PFDs) that function as prions *in vivo* and three negative controls that fail to exhibit this phenotype, all them sharing Q/N content with the Sup35 PFD [15]. pWALTZ correctly classifies the prion activity of these s-PFDs and controls. The score of synthetic PFD fit well with those of the PFD of natural yeast prions (Table 2). Thus, the differential prionogenicity of these artificial sequences correlates with the potency of their identified amyloid cores, suggesting that their prion propensities depend on their relative ability to form ordered assemblies nucleated by relatively short stretches displaying specific sequential properties and not in more diffuse and essentially sequence independent protein features spread out over large domains, as previously suggested [15].

**Table 2:** Prediction of the prionic behaviour of synthetic yeast prions.

Gene Name	pWALTZ window	pWALTZ Score
s-PFD1	NGEQSFWYQQNNNLQQQGNYQ	**74.73**
s-PFD2	QNQNGYYNNQNQIQQAQQNTQ	**74.36**
c-PFD1	LAMNQHTKLNNENNSQDFLQQ	65.44
c-PFD2	QMNKRYNKKYSSNHTQQTSNH	66.73
c-PFD3	AGQALQHQNHKRYENNQAWEQ	66.30

Synthetic PFDs (s-PFD) exhibiting prion behaviour in yeast and negative control sequences (c-PFD), as designed by Toombs et al. [15], where analysed with pWALTZ. Sequences with pWALTZ score >73.55 are considered prion-like. Predicted prion and non-prion sequences are shown in bold and plain text, respectively.

The number of human proteins containing sequence stretches resembling in composition to yeast PFDs account for ∼1% of the human proteome [32], [33]. The function of these domains remains unclear, however RNA- and DNA-binding proteins are enriched among human polypeptides containing putative PFD [32]. The Fus, TPD-43, hnRNPA1 and hnRNPA2/B1 ribonucleoproteins, all linked to neurodegenerative disorders, are included in this group. Despite the scoring of hnRNPA1 and hnRNPA2 fall below the cut-off for prion identification in yeast sequences using pWALTZ and also in composition based algorithms like PAPA, it has been recently reported that discrete missense mutations in the PFD of hnRNPA1 (D262V and D262N) and hnRNPA2 (D290V) cause multisystem proteinopathy and amyotrophic lateral sclerosis [34]. Importantly, these residues are located at the highest-scoring 21 residues window as identified by pWALTZ for both hnRNPA1 (G247-N267) and hnRNPA2 (Y275-N295) PFDs and all the pathogenic mutations increase the pWALTZ value (Table 3), suggesting that increased amyloidogenicity might account for the accumulation as cytoplasmic inclusions of the mutated species in animal models [34]. This suggestion is in line with the highest *in vitro* aggregation propensity of the disease-linked variants and the highest amyloid propensity of hexapeptides including the mutated residues, relative to those of the wild type sequences [34]. Moreover, hnRNPA2 and hnRNPA1 variants lacking the 287–292 and 259–264 sequence stretches, respectively, both inside the pWALTZ best scoring windows for the wild type proteins (Table 2), are aggregation resistant, even in the presence of preformed homologous wild type or mutant amyloid fibrils [34]. Accordingly, the new high scoring sequences in this deleted PFDs display significantly lower pWALTZ values than those in their respective wild type sequences (Table 3). Despite their aggregation properties, it cannot be affirmed that hnRNPA1 and hnRNPA2 or their mutants constitute *bona fide* prions in humans, since their propagation has yet not been demonstrated. Nevertheless, this property can be approximated exploiting the modular nature of yeast prion proteins. Kim and co-workers replaced the Sup35 nucleation domain with the core PFD from either hnRNPA2 or the D290V mutant and expressed these fusions as the unique copies of Sup35 in yeast cells. In agreement with its classification as a prion domain according to pWALTZ (Table 3), only the mutant variant can substitute for the Sup35 nucleation domain in supporting prion formation and specifically promoting the nucleation activity [34].

**Table 3:** Amyloidogenic regions in the PFDs of hnRNPA1 and hnRNPA2.

Gene Name	pWALTZ window	pWALTZ Score
hnRNPA1	247-GFGNDGSNFGGGSYNDFGNYN-267	68.48
D262V	247-GFGNDGSNFGGGSYNVFGNYN-267	71.82
D262N	247-GFGNDGSNFGGGSYNNFGNYN-267	71.32
Δ259-264 (a)	241-SGDGYNGFGNDGSNFGGGNYN-267	65.56
hnRNPA2	275-YDNYGGGNYGSGNYNDFGNYN-295	71.82
D290V (b)	275-YDNYGGGNYGSGNYNVFGNYN-295	75.03
Δ287-292 (a)	271-YGGGYDNYGGGNYGSGNYNQQ-297	64.66

The highest pWALTZ scoring windows and the pWALTZ values for these sequences are shown for the wild type, pathogenic and deleted variants of the proteins. Positions in which natural mutations occur are shown in bold, deleted regions in Δ mutants are underlined.

(a) Variants with reduced amyloid propensity relative to the wild type sequence.

(b) Variants that can substitute for the Sup35 nucleation domain in supporting prion formation and promote the nucleation activity in yeast.

A reason for the accuracy of the pWALTZ can be found inspecting the values for Q and N in the position specific scoring matrix (PSSM) behind Waltz: N is favourable for amyloid formation in all hexapeptide positions except position 5, and Q is favourable in all positions except 4 and 5 [16]. Therefore a sequence stretch with high Q/N content, in which position 4 is an N and position 5 is an amyloid-promoting residue, will have a strong amyloid forming potential. As position 5 is the most restrictive position in the Waltz PSSM, that leaves Ile, Phe and Tyr as the main options, of which the latter is a residue that occurs with high frequency in prion sequences. These considerations suggest that low complexity sequences biased towards Q and N might display an intrinsic propensity to accommodate one or more amyloid cores. However, because the amyloid propensity of Q and N is generally lower than the one of hydrophobic residues it is conceivable that, in order to nucleate the self-assembly reaction the amyloid cores in Q/N rich sequences should involve more residues than in typical amyloids were they tend to be very short and typically highly enriched in hydrophobic residues [12]. This would explain why predictions aimed to identify these very short and highly potent protein segments fail to classify correctly Q/N based prionic sequences [15] and why pure polyQ sequences require very large stretches to attain amyloidogenic potential [35].

### The relationship between aggregation and structural order in prion proteins

**Figure 3:**
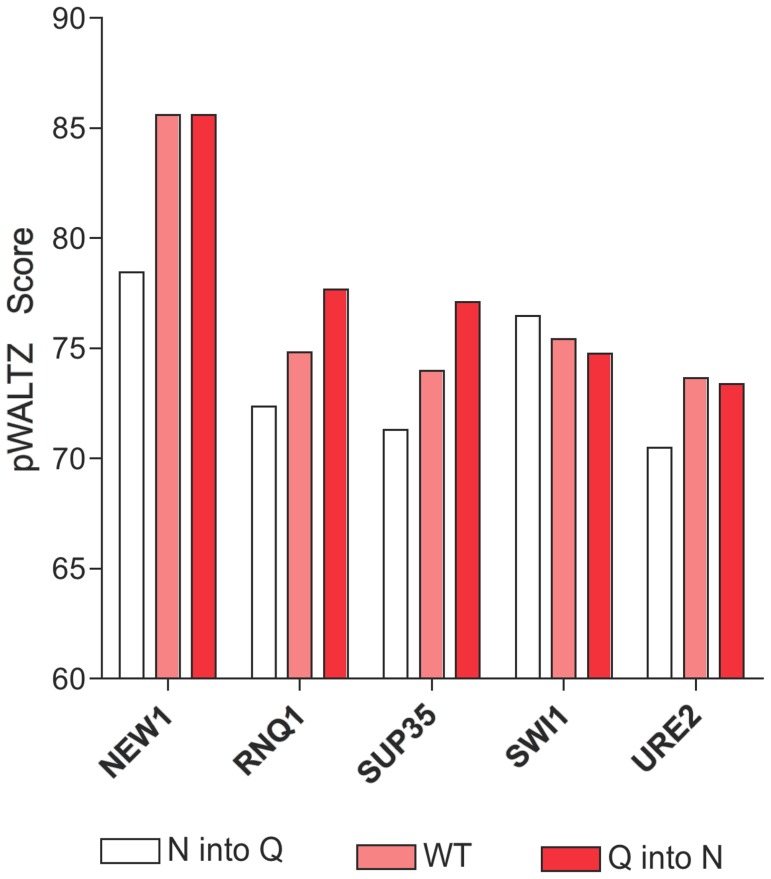
Amyloid propensities of Q and N residues in the context natural yeast prions. pWALTZ scores of wild-type (WT) (pink) and virtual mutants in which all Q residues are changed to N (red) or all N residues into Q (white) in the PrD of natural yeast prions.

A surprising finding in Alberti's study is that, despite differing only in a methylene group, the ratio of N to Q residues is an important determinant of the prion propensity of a sequence. Prionic domains are, as a trend, enriched in N whereas Q are more abundant in non-prionic sequences in their dataset [15]. Since according to our analysis amyloidogenicity seems to contribute significantly to prion-forming capability, it could be simply that N residues are more amyloidogenic than Q in the context of prion sequences, in agreement with the observation that according to the WALTZ PSSM, N is tolerated in more positions than Q in amyloid sequences (S1 Fig). Interestingly, the 21 residues long amyloid cores detected by pWALTZ in prionic sequences in our dataset contain an average of 9.9 N and 1.3 Q residues, whereas the equivalent sequence stretches in non-prionic sequences contain 4.1 N and 6.0 Q residues, respectively. Therefore, despite in both cases Q+N account for ∼1/2 of the residues in the core, amyloid cores in prionic domains are highly enriched in N residues, with a N/Q ratio of 7.9, whereas non-prionic cores display a N/Q ratio of only 0.7 at their cores. This suggests that despite their similar physicochemical properties these two residues endorse sequences with different amyloidogenic potential. Consistently, when we compared the predicted amyloidogenicity of known yeast PFD and of virtual mutants in which all N were replaced by Q and *vice versa* using pWALTZ, we found that, as a trend, the N to Q replacement decreases the amyloid propensity of the domains, whereas changing Q into N results in propensities similar that of the wild type sequence, when the core is already enriched in N residues, or increases the amyloid propensity of the domain (Fig. 3). These observations are in excellent agreement with the recent experimental demonstration by the Lindquist's group that N richness promotes assembly of self-templating amyloids whereas Q richness favours the formation of non-amyloid conformers [36].

**Figure 4:**
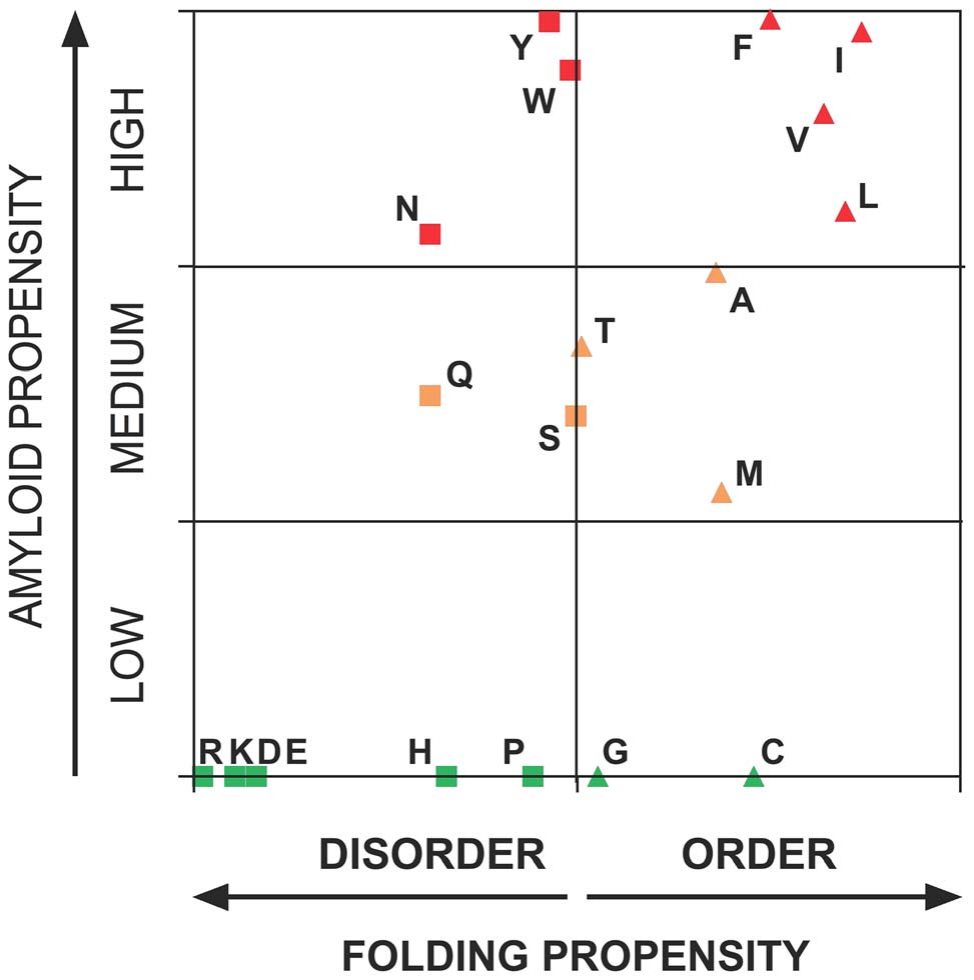
Relationship between amyloid and disorder propensities of natural amino acids. Ordered and disordered-promoting homo-polymeric amino acid stretches according to FoldIndex are represented as triangles and squares, respectively, and sequences with low, medium and high amyloid propensity as predicted by WALTZ are represented in green, orange and red, respectively.

**Figure 5:**
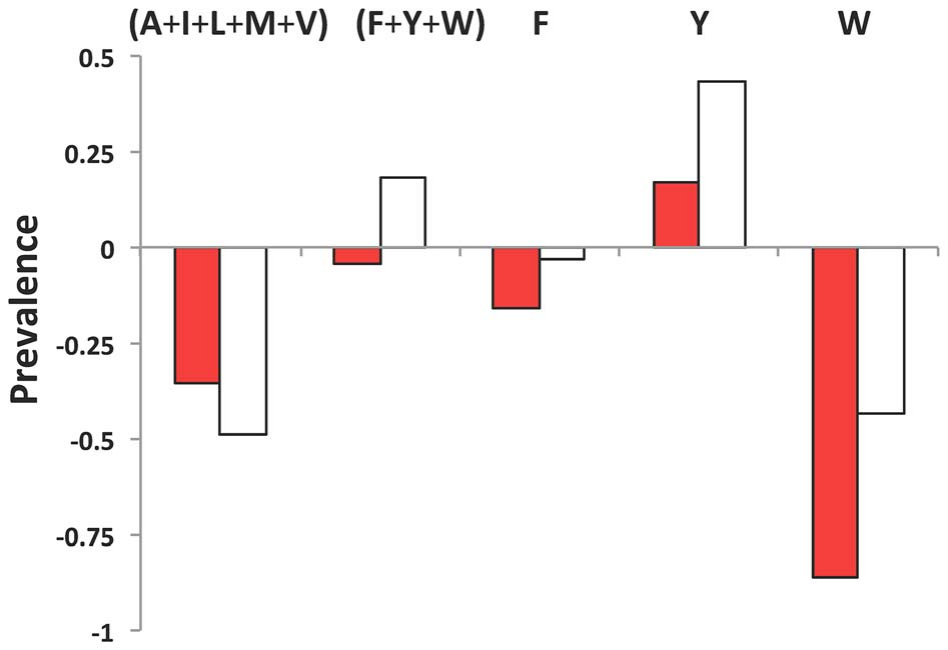
Hydrophobic residues in pWALTZ amyloid cores. The frequency of the indicated hydrophobic residues in pWALTZ amyloid cores relative to that in all the proteins in SwissProt is plotted for non-prion (red) and prion (white) domains. Positive and negative values correspond to overrepresented and underrepresented amino acids or amino acid groups, respectively.

The intriguing question thus remains of why PFDs are enriched in Q and especially in N and not in other residues with a higher hydrophobicity and/or β-sheet propensity, which would render them more amyloidogenic, as in typical amyloids. Together with their high Q/N content and their ability to form amyloid assemblies, an essential property of yeast PFD is that they lack regular secondary structure in their soluble state. It could be simply that Q and especially N constitute the most amyloidogenic residues that are still able to promote significant intrinsic disorder in a protein sequence. Not surprisingly, according to the FoldIndex algorithm [37] all the detected amyloid cores in prion domains are located in disordered protein regions. To test this possibility we constructed 21-mer homo-polymeric sequences for the 20 natural amino acids and analyzed their disorder and amyloidogenic propensity using Foldindex and pWALTZ simultaneously (Fig. 4). Interestingly enough, polyQ and specially polyN display both a high disorder and amyloidogenic propensity. Thus, Q and N enriched sequences would have a dual character that would allow them to maintain a certain amyloid potential and still remain disordered. However, polyQ and polyN render pWALTZ scores of 49.83 and 70.90, respectively, thus indicating that 21 residues core formed exclusively by these residues would not endorse prioneginicty to a sequence and therefore that the presence of hydrophobic residues in the core is a requirement for prion formation. The rest of predicted disordered sequences do not exhibit any amyloidogenic propensity according to pWALTZ, with the exception of polyY. Tyrosine is the most abundant hydrophobic residue in yeast PFDs [8]. We compared the composition of hydrophobic residues in the amyloid cores detected by pWALTZ in prionic and non-prionic sequences (Fig. 5) and found that non-aromatic hydrophobic residues are strongly underrepresented in both cores, relative to their average frequency in Swissprot [38], whereas aromatic residues are overrepresented in prionic cores and underrepresented in non-prionic ones, respectively. However, Tyr is the only residue that contributes to the overrepresentation of aromatic residues in prionic amyloid cores, being 2.7 times more abundant that the average in Swissprot (Fig. 5). It has been recently proposed that aromatic residues are favoured in PFD relative to non-aromatic hydrophobic residues, because they serve a dual function, promoting both prion formation and chaperone dependent prion propagation [39]. However, this does not explain why, despite differing in a single hydroxyl group, Tyr is much more prevalent than Phe at both the PFDs and their amyloid cores. A plausible reason for this bias is that Tyr displays a clearly higher amyloidogenicity/disorder ratio than Phe and the rest of hydrophobic residues, followed by Trp, which due to its size might cause steric hindrance in prion fibrillar structures, explaining its low abundance in PFDs [8]. Additionally, from the best scoring residues in the restrictive position 5 in the Waltz PSSM, Tyr is clearly superior in terms of disorder propensity, appearing thus as the best residue to endorse prionogenic Q/N rich amyloid cores with increased amyloid potential without disturbing significantly the PFDs disorder properties.

Long disordered regions in functional intrinsically disordered proteins (IDPs) are significantly depleted in N, whereas they are, for instance, enriched in Q or S residues [40]. No satisfactory explanation for this low representation of N residues in intrinsically disordered regions has been provided yet. Our analysis suggests that because, in contrast to prions, IDPs need to remain soluble during all their existence in the cell, the reduction in the proportion of N residues might respond to an evolutionary strategy to avoid the spontaneous self-assembly. The same reason might explain why the occurrence of homo-polymeric amino acid stretches of 20 or more Q in proteomes is four times higher than that of N repeats despite the abundance of these residues in the corresponding proteins is fairly similar (4.4 and 3.9% for N and Q, respectively) [41]. This may be especially true because mutation of one of several of the N residues in an N repeat into an hydrophobic residue might lead to the formation of a strong enough amyloid core to induce the protein aggregation.

The intermediate amyloid potential of N might be important for prion propagation. It has been shown that the yeast chaperone Hsp104 promotes prion propagation at intermediate concentration and that propagation can be blocked by both increasing as well as decreasing the level of this essential chaperone [42]. This is rationalised mechanistically by considering the balance between nucleation and growth of prion amyloids: when the chaperone breaks up amyloids without fully clearing them, the effective concentration of seeds is increasing. Fragmentation of fibrils has also been shown to determine the phenotype strength of different prion strains, as increased brittleness of fibrils results in a higher number of independently elongating fibril particles, increasing thus the efficiency of prion infection [43]. The presence of a strong and rigid amyloid core formed by a high proportion of highly amyloidogenic residues would reduce the fragmentation rate and accordingly, would decrease the prion propagation efficiency. In this context, N is likely the residue that provides the best balance between amyloid and propagation potentials.

## Discussion

**Figure 6:**
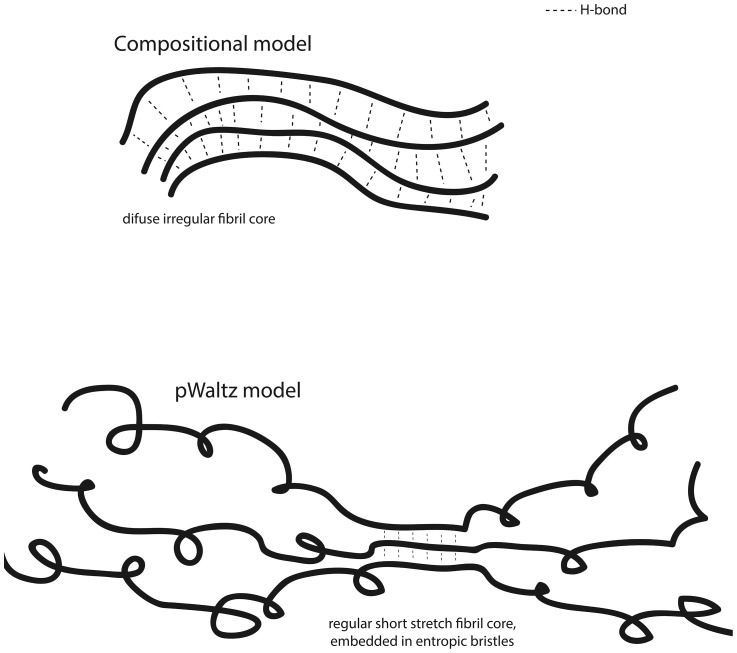
Alternative models for amyloid formation in prion-like domains. The compositional model relies on the establishment a large number of weak interactions whereas the pWALTZ model suggests a preferential nucleation by a short amyloidogenic stretch, whose amyloid propensity is modulated by the structural context.

The results in the present study together with several lines of evidence from the literature indicate that for a protein sequence to become a prion domain it requires: i) a specific region with significant amyloid propensity able to selectively nucleate the self-assembly into ordered, but brittle, amyloid structures ii) a disordered structural context that, in contrast to what happens in structured globular proteins, readily permits the domain self-assembly without a requirement for conformational unfolding, and iii) an amino acid composition that while allowing the domain to be soluble at the physiological concentrations required for the normal protein function still display a basal amyloid propensity, to which N residues would contribute significantly, promoting their self assembly in the presence of preformed amyloid seeds or when its concentration is increased. All these prion properties are readily predictable, opening an avenue for the accurate identification of prionic sequences in proteomes. Moreover, because aggregation of human PFD containing proteins might contribute to the etiology of a number of degenerative diseases, the present approach might find application in the detection of pathogenic genetic mutations associated with these disorders. Composition based methods provide good prion prediction accuracies by assuming that in these particular proteins amyloid formation relies on the establishment a large number of weak interactions between side-chains in long disordered domains (Fig. 6), but in fact they mask the presence of specific short sequence stretches that, as in other amyloids, would trigger the conversion of prions from the soluble to the aggregated and transmissible state (Fig. 6). Instead of two contrasting views, our analysis suggests a model for Q/N rich prions, where a classical amyloid core is embedded in a compositional context that reduces the amyloid nucleation potential, giving rise to sequences that are strongly dependent on seeding.

## Materials and Methods

### Selection of putative prion sequences

From the 100 candidates putative PFD sequences in Alberti's data set [8] only those candidates for whom all four experimental assays could be carried out were selected for further analysis, accounting a total of 83 sequences.

### Classification of prion and non-prion sequences

We used the combined accumulative score reported by Alberti et al [8]. For the formation of intracellular aggregates and Sup35 switching behavior, the putative prion sequences positive or negative proteins received 2 and 0 points, respectively. For SDD-AGE (48 hours of induction) and *in vitro* assembly assays, the candidates received points according the resulting scale: -  = 0 points, +  = 1 point, ++  = 2 points, +++  = 3 points. Therefore, the maximum combined score is: 2+2+3+3 = 10 [8]. Sequences scoring ≤2 and being positive in one assay at maximum were considered as non-prions and sequences being positive in all four assays and scoring ≥9 as prions (Table 1). SUP35 was not included in the test set to avoid overlap with the WALTZ training set.

### Sequence analysis with WALTZ

The Waltz prediction method described in Maurer-Stroh et al [16] is available at http://waltz.switchlab.org/. The algorithm allows selecting a custom threshold for sequence analysis. Low thresholds are useful to determine the aggregation propensity of sequences without position-specificity restrictions whereas high thresholds select sequences fulfilling the position-specific requirements for amyloid formation. In order to identify the more predictive threshold, receiver-operating characteristic (ROC) analysis of the WALTZ scores for the complete PFD sequences were obtained using thresholds ranging from 0 to 100%. The ROC curve is a graphical plot displaying the performance of a binary classifier system as its discrimination threshold is varied. It plots the fraction of true positives out of the total actual positives (sensitivity) vs. the fraction of false positives out of the total actual negatives (specificity), at various thresholds. The area under the curve (AUC) reflected the accuracy of the discrimination [44] and therefore the best balance between position-specific and non-specific aggregation prediction. The AUC estimates the statistical significance of the classification test and represents the probability that when a pair of positive and negative sequences is randomly selected from the pool, the WALTZ score will be higher for the positive one. The Youden's J statistic (J), also called Youden's index, a single statistic was used to capture the performance of the diagnostic test since it both measures the effectiveness of a diagnostic marker and enables the selection of an optimal cut off point for the marker, which corresponds to the best combination of sensitivity and specificity in the prediction (J =  Sensitivity + Specificity −1) [45]. The average accuracy was calculated as: Accuracy  =  (number of True Positives + number of True Negatives)/number of elements in the Total Population, for any given prediction. A pWALTZ executable file, sequence examples and use instructions can be freely downloaded for academic use at http://bioinf.uab.es/pWALTZ/. The order/disorder context of the detected amyloid cores was analyzed with FoldIndex [38] using the default 51-aa window size along the complete PFDs.

## Supporting Information

S1 Fig **Amino acid preferences in the WALTZ scoring matrix.** Residues log-odd scores for amyloid core formation in a given hexapeptide position.
(TIFF)

S2 Fig **Example of pWALTZ procedure for scoring prion and non-prion sequences.** The putative PFDs of ENT2, MCM1 and SWI1, scoring 0, 0 and 9, respectively, according to *Alberti et. al.* scale of prion propensity (Cell 2009 137, 146–158) are analysed. In agreement with experiments, ENT2 (below the threshold) and MCM1 (pWALTZ score  = 59.63) are predicted as non-prions and SWI1 (pWALTZ score  = 75.43) as containing a prion domain.
(TIFF)

S1 Table **Amyloid prediction of sequential variants in the 39 to 46 positions of the Sup35–27 PFD.** PSI^+^ and PSI^-^ correspond to 8 residues stretches able to support or not prion conversion when substituting the original sequence in the Sup35–27 variant, respectively. As described by as Toombs and co-workers (Mol Cell Biol. 2010 30(1): 319–32). Sequences predicted to be amyloidogenic by WALTZ using the default parameters are shown in bold.
(PDF)
