# Supplementary figures and images for: What Makes a Protein Sequence a Prion?
Source: PLoS Comput Biol. 2015 Jan 8;11(1):e1004013. doi: 10.1371/journal.pcbi.1004013 (PMC4288708; doi:10.1371/journal.pcbi.1004013)

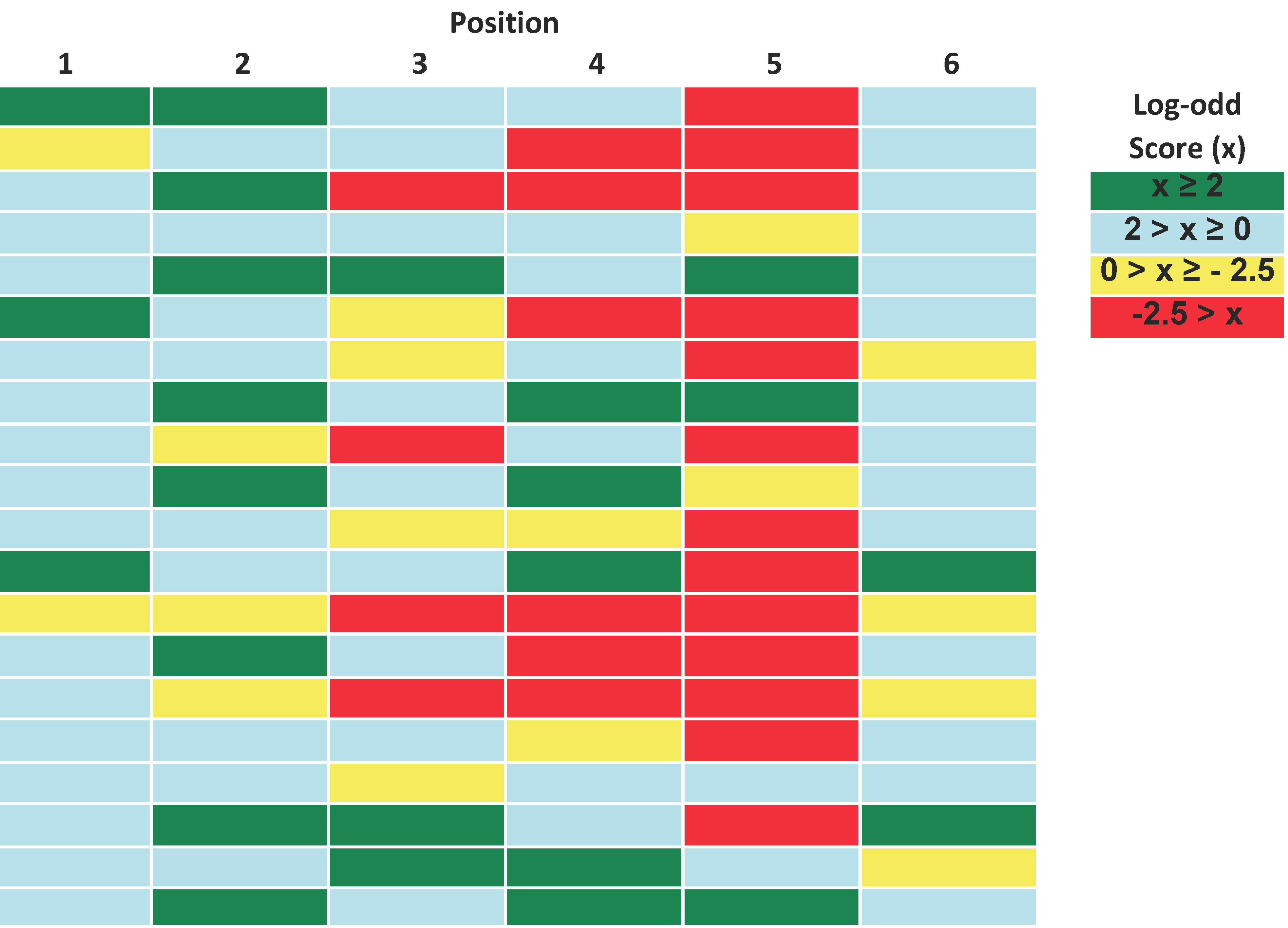

Supplement: S1 Fig — Amino acid preferences in the WALTZ scoring matrix. Residues log-odd scores for amyloid core formation in a given hexapeptide position. (TIFF) [file pcbi.1004013.s001.tiff]

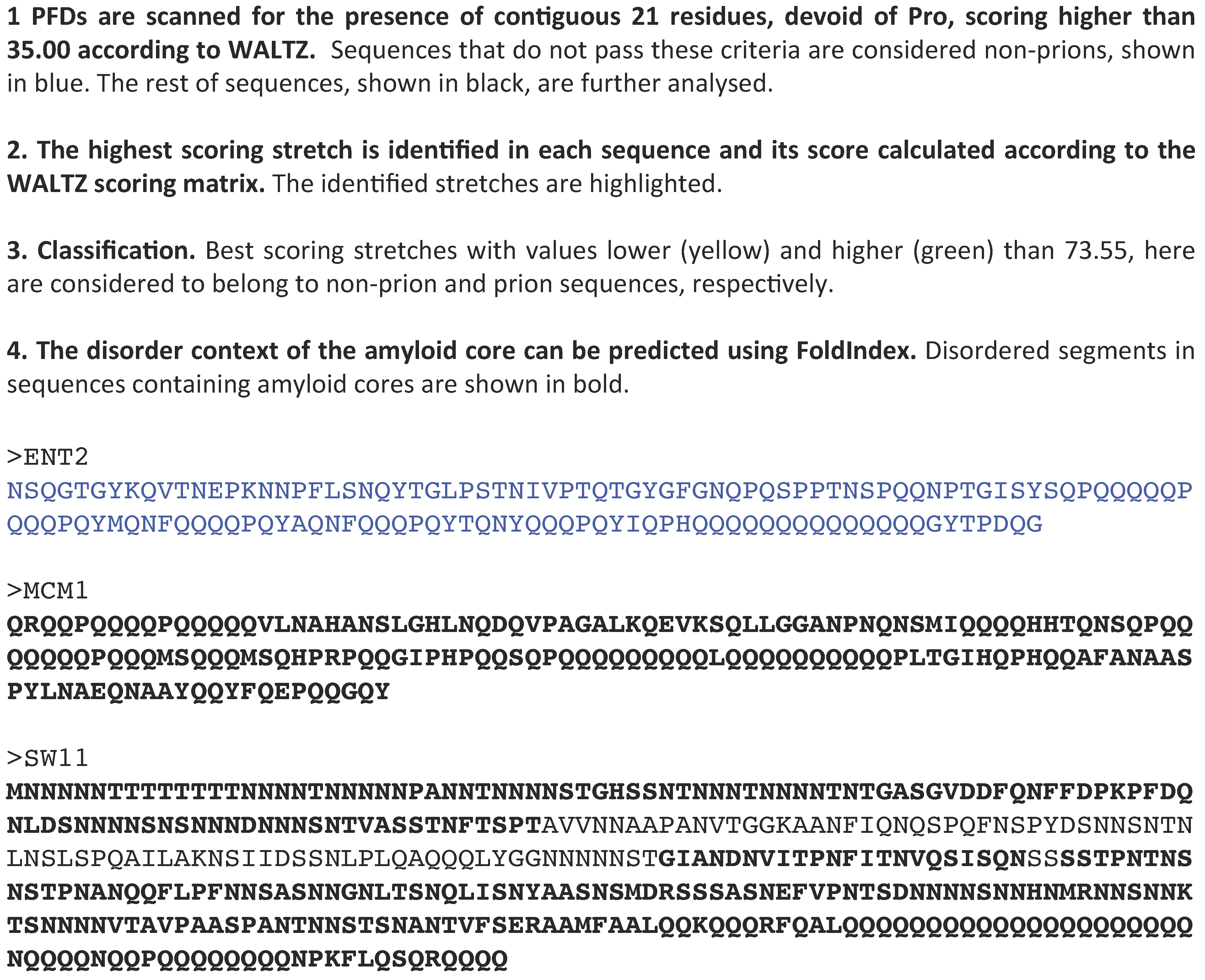

Supplement: S2 Fig — Example of pWALTZ procedure for scoring prion and non-prion sequences. The putative PFDs of ENT2, MCM1 and SWI1, scoring 0, 0 and 9, respectively, according to Alberti et. al. scale of prion propensity (Cell 2009 137, 146–158) are analysed. In agreement with experiments, ENT2 (below the threshold) and MCM1 (pWALTZ score = 59.63) are predicted as non-prions and SWI1 (pWALTZ score = 75.43) as containing a prion domain. (TIFF) [file pcbi.1004013.s002.tiff]
