# Supplementary material for: What Makes a Protein Sequence a Prion?
Source: PLoS Comput Biol. 2015 Jan 8;11(1):e1004013. doi: 10.1371/journal.pcbi.1004013 (PMC4288708; doi:10.1371/journal.pcbi.1004013)
Supplement: S1 Table — Amyloid prediction of sequential variants in the 39 to 46 positions of the Sup35–27 PFD. PSI+ and PSI- correspond to 8 residues stretches able to support or not prion conversion when substituting the original sequence in the Sup35–27 variant, respectively. As described by as Toombs and co-workers (Mol Cell Biol. 2010 30(1): 319–32). Sequences predicted to be amyloidogenic by WALTZ using the default parameters are shown in bold. (PDF) [file pcbi.1004013.s003.pdf]

Table S1. Amyloid prediction of sequential variants in the 39 to 46 positions of the Sup35-27 PFD. PSI$^+$ and PSI$^-$ correspond to 8 residues stretches able to support or not prion conversion when substituting the original sequence in the Sup35-27 variant, respectively. As described by as Toombs and co-workers (Mol Cell Biol. 2010 30(1):319-32). Sequences predicted to be amyloidogenic by WALTZ using the default parameters are shown in bold.

PSI$^+$	PSI$^-$
**VNIFPYYN**	**CKSVCNFD** TDPWVPHP
**VTSGSYNT**	**ADSASNAS** AQDSHPDI
**AHTTNMIV**	**ICWHTEPY** NPEVPNAN
**LLVHSNAI**	**NNPQYLFK** THHSHTLP
**HSNVSVIH**	**GANSAITN** DERPWCPE
**TRIWNFSG**	**SVNPALYR** TAYVRHID
**GSLSLQYF**	**CFLRSYMG** YLPFMDTP
**MLSSNFIH**	**NISPFSKD** GPTMNNRD
**SSGPLNFI**	**KMTTNTKH** PPIVKPRT
**QFVARVFR**	THRHNKHR VDDRHMFS
**LKSVITWN**	KGSPSTPT EAPSKSAQ
**SVHVNSTS**	GISTRSQE RPERRSNP
ASNIVMNC	QVASQNGR VSLSKNRL
YNCSVNML	LRDPDTCS CIKHINSI
FSIYMPYK	RKATDLFP PVPSSSQP
WGARQFNI	DRYKGKPH SHLWRRNR
VTTDILAM	DPNAALVF DSHTGTPR
RRDYLTRF	HIHPLFIH STVPPPHH
STVICGVI	TLARRDPP VNCARGTA
IHFWPRAP	PNASGIHY SSNKFMHT
TWAPIMVY	NGPAYPLA GFTKALPG
MFQHGIGV	ALSSRQWS SGVSTAVR
YHSVEFRI	IDKNLMSH LNRITLRN
TTVNHHFN	IVPRNVNC VALIPKTA
IFDIANHS	HNLANHSH MTQNPHIF
LQPCYCSR	LSARPLGH LGNPTFHY
CLSPAECR	
